# Efficiency evaluation of two estrus synchronization protocols in estrus response and conception rate of dairy cows in the Dalocha district, Ethiopia

**DOI:** 10.1016/j.heliyon.2022.e12781

**Published:** 2023-01-04

**Authors:** Sharew Mekonnen Haile, Belete Kuraz Abebe, Tigst Wendala Tesfa

**Affiliations:** Department of Animal Science, Werabe University, P.O.Box 46, Werabe, Ethiopia

**Keywords:** Alfagladin C (Cloprostenol), Conception rate, Estrus response, Estrus synchronization

## Abstract

The study aimed to determine the effectiveness of single and double-shot estrus synchronization protocols on conception and estrus response rates in dairy cows. Among 195 sampled female animals, only 174 cows and heifers met a standard of the protocols. Animals were prepared for hormone injections based on their breed, parity number, and body condition score (BCS). Among 174 sampled animals, 120 were indigenous and the rest 54 were crossbreds, and 143 cows and 31 heifers were selected for single alfagladin C (Cloprostenol) injections. Whereas, 16 of them were given double alfagladin C (Cloprostenol) injections when they did not showed estrus prior to single injection. All data was collected and analyzed by SPSS version 20 software. Through observation, inspection and rectal palpation, 90.8% of 174 synchronized animals showed estrus response with a single dose, whereas 16 of them (100%) showed estrus response with double injections. Overall, 48.3% of cows and heifers became pregnant. The conception rate of cows/heifers varied significantly through each protocol, with 50% of animals conceived by double injection treatment and 48.1% of cows conceived through single shot treatment. Additionally, there was a substantial difference in estrus response across breeds, BCS, and parity number. Furthermore, the rates of conception in local and crossbred cows were 40.8 and 64.8%, respectively. In terms of body condition, a high conception rate (58.6%) was observed in the good body condition score. The conception rate and estrus response were significantly different in parity number. In general, the double protocol outperform than the single protocol in terms of estrus and conception rate. Accordingly, stakeholders or artificial insemination technicians might apply double protocols after a single injection to obtain remarkable results. Nevertheless, strict follow-up is required, and more resources at the farmer's management level are required.

## 1 Introduction

Estrus synchronization (ES) and artificial insemination (AI) continue to be a major influential technologies for cattle producers in terms of genetic improvement, reproductive management, and animal reproductive and productive performance [1]. ES entails manipulating or controlling females' estrous cycles in order to breed them at roughly the same time [2]. ES and its various protocols are useful tools in a cow herd's reproductive management, but they require optimal diet, good body condition, and health, as well as experienced manpower. Inadequacy in any of these areas, on the other hand, can spell disaster for these technologies [3,4]. Consequently, all synchronization protocol techniques necessitate proper management, regular estrous cycles, and cows in good physical condition, as well as attention to detail and adequate feed [5].

A nutritional state, improper management, ovarian alterations, endocrine events, and even uterine infection may all play a role in the varied results acquired after hormone injection by different professionals. However, a number of factors influence both the reproductive and productive performance of local (Zebu) and crossbred cattle, including the poor performance of Holstein Friesian cattle in the tropics. The main causes of failure of the technology are poor nutrition, lack of effective estrus diagnosis, artificial inseminators and breeding control problems, illness, calf suckling, then quiet heat [6,7]. In the tropics, majority cows, plus hybrids, unsuccessful to calve every 12–13 months afterward the initial calving, much as they do on smallholder farms [8]. Unsuccessful of appropriate heat determination was the well-known difficult in tropical small-holder farmers' breeding programs, causing in a decreasing of lifetime milk yield, reduction in the figure of calves born per life time, extreme days open, and rise in reproductive culling [6].

According to Aulakh [9], incapability to expect the duration of heat for individual cows or heifers in a group makes it unreasonable to practice ES and AI due to labor mandatory for determination of heat; proper control of a time of estrus is difficult; most of the time, peak heat activity happens during nighttime, and determining the standard onset of standing heat is problematic deprived of 24-h observation. Visual inspection is the most commonly employed technique of heat recognition for cow breeding, which renders heat detection poor in most dairy farms [10]. Because visual detection is a less efficient method, cattle are frequently left unnoticed when they come into estrus. At time of cows enter into heat in the evening, while people are less busy and prefer to rest, the situation gets more serious. Similarly, cystic ovarian disease causes a longer calving gap by altering the animal's regular estrus cycle [11]. As a result, lengthy postpartum anestrus periods are a well-known problem in cows raised in tropical environments. Consequently, ES and its derivative protocols are the main backbone for solving the above-listed problems [12].

PGF2 alpha is one of the ES protocols and it is a naturally occurring hormone that produces luteolysis (regression of the corpus luteum) and decreases progesterone output, resulting in a return to estrus. For example, PGF2 alpha is only used in estrous-cycle cows to synchronize an ovulatory estrus, and injection of PGF2 alpha into pre-pubertal heifers and anestrous cows are ineffective because of lack of luteal tissue. Additionally, PGF2 alpha injection may not cause non-cycling cattle to begin cycling [13]. As a result, before beginning treatment with PGF2 alpha alone to estrus synchronize, PGF2 is critical towards determine the percentage of cycling heifers or cows. Most efficient estrus synchronization techniques combine treatment with a progestin and administered of PGF2 alpha in herds with both cycling and non-cycling females. PGF2 alpha is particularly successful by inducing abortion in pregnant nutritional heifers earlier 100 days of gestation. There are two types of PGF2 alpha-based protocol injection: single-shot injection and double-shot injection [13].

Most of the time, smallholder farmers can evaluate the efficacy of ES followed by AI service technology; solely via conception rate and calf production. A more realistic estimate of 50–55%, on the other hand, is more accurate [4,14]. Recently, the Ethiopian government and other non-governmental organizations (NGOs) have been working on improving dairy productivity through breed development initiatives through the use of ES and AI technology [15].

In Ethiopia, only a little research has been conducted on the efficacy of ES employing single and double-shot PGF2 alpha-based protocols followed by AI application. Hence, it is insufficient to verify that the causes of failure as expected by farmers are either due to method synchronization or artificial insemination methods, farmers' perception of estrus detection, semen quality, dairy herd management systems, and breed types [14]. Moreover, there has been no investigation done to know the efficacy of single and double shot injections of PGF2 alpha-based protocol followed by AI application on level of pregnancy in dairy cows in the study district so far. Consequently, this study was aimed to evaluate the efficacy of a combination of single and double-shot PGF2 alpha injections followed by AI application on pregnancy and estrus response rate of dairy cows kept in Dalocha district, Silte Zone, with the following specific objectives: a) To determine the efficacy of single and double estrus synchronization shot protocols on dairy cow estrus response and pregnancy rate. b) To identify the effect of hormones on breeding, pregnancy, and estrus response rate. c) To examine the association of hormones and body condition scores on conception and heat response rate. d) To identify the association of hormones and parity number on gestation and estrus response rate.

## 2 Methodologies

### 2.1 Description of dalocha district

**Fig. 1:**
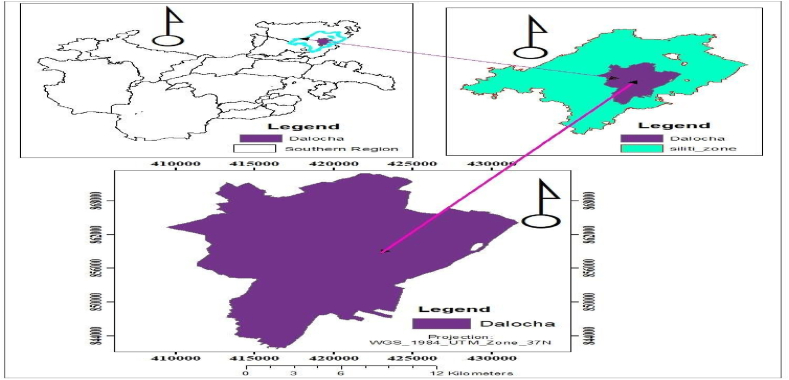
Dalocha district location map.

The study was conducted in Dalocha district, in Silte zone of Southern Ethiopia, from October 2020 to September 2021. The district is 14 km away from the Silte zone. Silte zone has different agro-ecological zones ranging in altitude between 1500 and 3700 m. a.s.l. namely highland (Dega) 20.5% and midland (Woynadega) covering 79.5% of the zone. The mean temperature range from 12 to 26 0C and the average annual rainfall ranges from 780 to 1818 mm. Wheat, Maize, Sorghum; and Enset are the major crops (WBOA 2010). Dalocha is located at latitude of 7° 44′ 59.99″ N and a longitude of 38° 19′ 60.00″ E. The mean temperature and altitude of Dalocha district were 12–22.5 °C and 1900_m.a.s.l) respectively Fig. 1(https://latitude.to/articles-by-country/et/ethiopia/256155/dalocha-woreda).

### 2.2 Experimental animal selection

A total of 195 indigenous and crossbred (<75 exotic blood level) cows and heifers were used in this study, with only 174 cows and heifers confirming the estrus synchronization criteria being picked by AITs from farmers. The BCS (Body Condition Scoring) system was used, which ranged from 1 to 5 (1 = a very emaciate cow, to 5 = highly fat cover cow; but for this study ideal body condition scores system was used which fall in the range of (2.5–4). General health condition, existence of mature corpus luteum, and normality of reproductive system were investigated. A pregnancy test using rectal palpation was performed before hormone delivery to prevent abortion. Synchronized cows and heifers were maintained with straw and locally available green grasses. Regular monitoring of cows' estrus response and health was performed by the local veterinary, AIT, and researchers.

### 2.3 Study design and sampling procedure

Purposive and random sampling approaches were used to choose the study subjects. The primary unit was the availability of dairy herds in specific villages. Using a simple random selection procedure, an individual sample was chosen from the previously identified dairy herd. Healthy animals were considered for treatment. Experimental animals were randomly allocated to a single injection (single shot alfaglandin c (Cloprostenol)). On the other side cows and heifers were not come in to heat in single injection on Day 1–5, they allocate in double protocol and re-injection of 2 ml alfagladin C (Cloprostenol) per animal intramuscularly after 11 days. The conception rate is calculated by dividing the total number of inseminated cows or heifers at a given time by the proportion of pregnancies confirmed by rectal examination at day 90–120 of post insemination.

#### 2.3.1 Single-shot PGF2α synchronization method

Rectal diagnosis and animal observation were used on returning cows and heifers who met the standard for this program. Animals were examined for any external signs of estrus. So far, five days, all cows and heifers have been seen 4 times per day: morning, midday, early evening, and midnight. Cows and heifers that met all of the preconditions were given a single injection of 2 ml of Alfagladin C (Cloprostenol) intramuscularly (IM) on Day 0 and then inseminated 1–5 days later when they were only exhibiting estrus activity.

#### 2.3.2 Double-shot PGF2α synchronization method

In this method, cows and heifers did not come into heat in a single injection on Day 1–5. They were allocated to a double protocol and re-injection of 2 ml of alfagladin C (Cloprostenol) per animal intramuscularly after 11 days. Cows and heifers were detected for heat expression after 24 h of the second injection of alfagladin C (Cloprostenol) then bred 6–12 h post estrus approved.

#### 2.3.3 Study population and their management

Cows and heifers with good body condition scores, good health with no performance anomalies of the genetalia organs to validate the reproductive stage, and developed corpus luteum were used in the investigation. Rectal palpation was used to do a pregnancy test prior to hormone delivery.

### 2.4 Data analysis

Using Microsoft Excel® 2010 (April 15, 2010) the data collected from cows and heifers' reproductive performance was categorized, filtered, and coded thereafter transferred to SPSS version 20 (2011) for analysis using cross tabulation at a significance level of α < 0.05. The results were given as descriptive statistics in percentage tables. The influence of parity, BCS, breed, and other factors on pregnancy and estrus response rate were analyzed using descriptive statistics.

This study used models for the analysis of the conception and estrus response rate data as follows:

Yijlm = Yijl = μ +wi + dj + pl+a +bn + eijln

Where, Y = the response variables Pregnancy (positive or negative) and estrus response.

μ = Overall mean

wi = Fixed effect of ith prostaglandin shooting protocol (single and double).

dj = Fixed effect of jth breed (j = indigenous, cross bred <75%exotic blood level).

pl = Fixed effect of lth parity number (0, 1,2,3,4,5and 6 parous).

bn = The effect of nth body condition score of cows on pregnancy rate (Good Moderate and Poor with the range of 2.5–4).

eijklm = residual error.

### 2.5 Ethical consideration

Before any attempt to perform the experiment and, the protocol was approved by werabe university research and ethical board committee.

## 3 Results and discussion

### 3.1 The effect of alfaglandin C hormone on estrus response and conception rate

#### 3.1.1 Estrus response and conception rate per breed

The current finding of the heat response rate in local and cross-breeds was 86.7 and 100%, respectively (Table 1). This result demonstrates that the estrus response differs significantly between breeds (p < 0.03). This implies that it is more successful in cross-breeds than in local breeds. This difference per breed of cow might be because of the period of estrus in exotic breeds is higher, as a result response to hormone treatment is advanced. In addition to this; the variation is due to the difference in temperature between the surroundings, feed, and the feeding method. The current finding result is contradicted with the results of reported by Mwanza [17], who found that 34% of crossbred cows and 14% of local cows were respond. The present finding of an estrus response rate was in line with the study of Tegegne et al. [18], who stated that 97.7% of them were respond and 100% in Adi-grat-Mekelle; and Gebrehiwot et al. [19], who described that 92.17% of them were responding to the test. On the other hand this study result was, closely analogous with the results of [20], which recorded 87.9 and 86.9% estrus response rate in native and crossbred cows, respectively.

**Table 1:** Estrus response and pregnancy rate across breed.

Parameter	Breed type			Total	p-value	
	Local		Cross (<75%)			
Estrus response rate rowhead						
Not responded (N)	16		0	16	0.03	
%	13.3		0.0	9.2		
Responded(N)	104		54	158		
%	86.7		100	90.8		
Total(N)	120		54	174		
%	100		100	100		
Bcvn rowhead						
PD^+^(N)	49	35		84		0.03
%	40.8	64.8		48.3		
PD^−^(N)	71	19		90		
%	59.2	35.2		51.7		
Total	120	54		174		
%	100	100		100		

N = number of cows and heifers, PD^+^ and PD^−^ = pregnancy positive and negative respectively.

In terms of conception rate, 48.3% of hormone-injected cows and heifers conceived, and both regimens produced 84 calves (Table 1). In contrast to Ref. [21], who reported (64%) in native cattle and (57%) in Holstein Friesian crosses, cross breeds had a higher conception rate than local breeds (Table 1). This difference might be due to the animals' genetic makeup, body condition, and management practices. The current finding is subsidiary compared to the finding of Tegegne et *al.* [18] that ascribed 57.7 and 61.7% of conception rate.

#### 3.1.2 Estrus response and conception rate per body condition score (BCS)

In accordance with [22], who reported 98.3% in good, 90.3% in optimum, and 47.8% in low BCS cows and heifers, the highest conception rate was recorded in this study in good body condition, followed by moderate and poor body condition score cows and heifers (Table 2). This result demonstrates that the effect of Alfaglandin C hormone had a significant effect on the conception rate of cows and heifers with good BCS and ES performed on animals with inadequate body condition score led to reduced heat sensitivity and pregnancy rates [23–25] According to Ref. [24], cows and heifers with 3.5–4 BCS had a substantially greater (P < 0.05) pregnancy rate (56.5%) than cows with 2.5–3 and 4.5 BCS. The study conducted by Ref. [25] observed a difference in pregnancy rates among cows of different BCS groups. Providing a well-balanced diet will aid in the development of healthy BCS; resulting in a high pregnancy rate [24]. Higher pregnancy rates in cows with good BCS are documented by Ref. [26] in Bangladesh. Nutrition can change gonadotropin secretion and cows with BCS of less than three had reduced pituitary responsiveness to GnRH [27].

**Table 2:** Estrus response and conception rate per BCS.

Parameter	BCS				Total	P value
	Good	Moderate	Poor			
Estrus response rate rowhead						
Not responded (N)	2	9	5		16	0.006
%	1.7	20	38.46		9.2	
Responded (N)	114	36	8		158	
%	98.3	80	61.54		90.8	
Total (N)	116	45	13		174	
%	100	100	100		100	
Conception rate rowhead						
PD^+^ (N)	68	5	11	84		0.00
%	58.6	38.5	24	48.3		
PD^−^ (N)	48	8	34	90		
(%)	41.4	61.5	75.6	51.7		
Total (N)	116	13	45	174		
(%)	100	100	100	100		

N = number of cows and heifers, PD^+^ and PD^−^ = pregnancy positive and negative respectively.

#### 3.1.3 Estrus response and conception rate per double and single shot protocol

In the present study, 90.8% of hormone-injected cows and heifers responded from days 3–5, whereas 9.2% of cows and heifers did not respond in the single shot protocol. Out of 16 double injected cows, all of them (100%) were showed estrus response. The effect of alfaglandin C on conception rate in the double and single shot protocols was statistically significant (p < 0.05), which means almost half of the cows (48.1%) became pregnant in single injection protocols (Table 3). This finding is close to the study done by Ref. [28] in the same protocol, although it contradicts those reported by Ref. [29] at 67% of the conception rate in the double injection protocol. This discrepancy could be attributable to cattle management practices, body condition score, parity number of the animals, and artificial insemination technician efficiency. The current result is greater than the 32.17% pregnancy rate reported by Ref. [19] in Wukro Kilte Awulaelo district, Ethiopia. The current finding of the conception rate in single injection was (48.1%) more advanced than the 13.7% reported by Ref. [30] in the Bahir Dar milk shed area, Ethiopia. In this study, conception rates in single and double injections were 48.1 and 50%, respectively. This finding is consistent with the findings of [28], who reported a 50% pregnancy rate in single injection and a 60% pregnancy rate in double injection in the north Shewa zone of Ethiopia, and [31], who reported a 55% conception rate.

**Table 3:** Estrus response and conception rate protocol.

Parameter	Shot protocol type		Total	P value
	Double	Single		
Estrus response rate				
No response (N)	0	16	16	0.043
%	0.0	9.2	9.2	
Response (N)	16	158	174	
%	100	90.8	90.8	
Total (N)	16	158	174	
%	100	100	100	
Conception rate per protocol				
PD^+^ (N)	8	76	84	0.005
%	50	48.1	48.3	
PD^−^ (N)	8	82	90	
%	50	51.9	51.7	
Total (N)	16	158	174	
%	100	100	100	

N = number of cows and heifers, PD^+^ and PD^−^ = pregnancy positive and negative respectively.

#### 3.1.4 Estrus response and pregnancy rate per parities

**Fig. 2:**
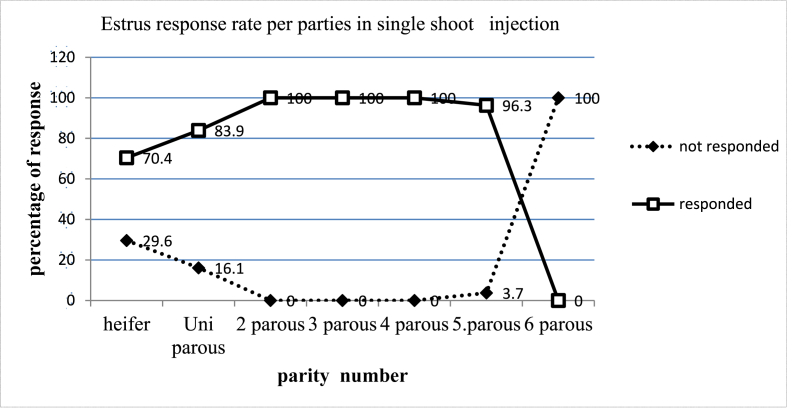
Estrus response rate per parity.

The current results show that alfaglandin C had a greater effect on estrus response in multiparous cows. The estrus response rate increased from heifer to two parous cows and remained steady or static from two parous to four parous cows (Fig. 2). After injecting alfagladin C (Cloprostenol) hormone, the estrus response rate of cows above five parous decreased, indicating that they had a lower estrus return rate than the remainder of the cows. In two, three, four, and five parous cows, the proportion of estrus response after a single injection of alfagladin C (Cloprostenol) hormone was 100, 100, and 96.3%, respectively. As shown in Fig. 2, alfagladin C (Cloprostenol) has a highly significant effect on parity numbers in general.

**Fig. 3:**
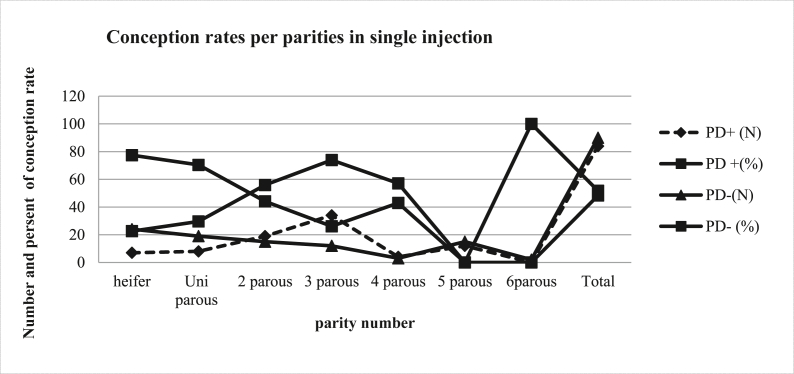
Conception rate per parity.

Fig. 3 depicts the conception rate per parity. According to the current study, the effect of alfagladin C (Cloprostenol) hormone on conception rate differed dramatically depending on parity. Two, three, four, and five porous conception rates were 55.9, 73.9, 57.1, and 44.4%, respectively. As a result, alfagladin treatment was more successful in two to four parous than in heifer, uni, five, and six parous. Out of the 174 inseminated cows and heifers 84 of them were conceived. Moreover, the total conception rate was 48.3% in percentage terms. As demonstrated in graph 2, 51.5% of cows and heifers were not conceived. In this study, the conception rates per parity were 55.9%, 73.9, 57.1, and 44.4%, two-five parous, respectively. This result is also in line with the findings of [28] in the Amhara region, Ethiopia. This data revealed that the conception rate was higher starting from 2 to 4 parous, then declined at 5 to 6 parous cows, which is consistent with [32] who found that the pregnancy rate increased with advancing parity starting two to sex, then after declined at parities seven and eight. The effect of parity in different synchronization methods is abundantly demonstrated by the findings of many investigations.

Nevertheless, the result should be dependent on dairy animal health and management practice [4,33]. Heifer, parity 1 and parity 5 cows had a considerably greater (P < 0.05) conception rate (57.1%) than heifers, parity 1 and parity 5. Similarly [21], found that cows at 2 and 3 parity had a greater conception rate than nulliparous cows. Previous research has shown that pregnancy rates differ depending on parity [34,35]. Cows in their first three parties had advanced pregnancy rates than cows in later parties. In addition [32], found that increasing parity from 2 to 6 resulted in a higher pregnancy rate, which then declined in parities seven and eight. Variables in conception rates between parities could be related to breed and environmental differences. The effect of Alfagladin C (Cloprostenol) on estrus response per parity in single shot injection was varied across parity numbers, which is better in parous 2–4 (Fig. 3). This is consistent with the findings of [28] in heifers and more than four.

## 4 Conclusion

This study was initiated to change farmers' negative attitudes or unwillingness toward ES and AI in dairy cow conception rates. The study was conducted to investigate whether single or double shot protocols were effective at increasing heat response and pregnancy rate. The findings also show that the percentages of pregnancy rate and heat response differed meaningfully across breeds, parity, and BCS. In general, cows and heifers given a double injection of Alfagladin C (Cloprostenol) responded better than cows and heifers given a single injection. The same is true in terms of the difference in conception rates between the two methods. Depending on the above result, we recommend the following a) Even if there were numerous challenging issues that contributed to the failure of estrus synchronization and the low conception rate, it is critical that this be performed by a community breeding program service. b) Due to the impact on estrus response and conception rate, it is preferable to use double injections after a single injection. The major limitation of this study was that it is difficult to re-inseminate after 5 days of synchronized cows or heifers in both protocols due to a lack of transport, the perception and skill gap of farmers on estrus detection, and the negative attitude of farmers toward this technology.

## Availability of data and materials

The original documents as well as raw data used to the analysis of this article are available in the hand of corresponding author for reasonable request.

## Funding

We thank Werabe University for funding this research through grant number WRU1312.

## Declaration of competing interest

All authors declare that no any opposing idea or interests.
